# Controlled molecular arrangement of easily aggregated deoxycholate with layered double hydroxide

**DOI:** 10.1098/rsos.230506

**Published:** 2023-10-11

**Authors:** Kyounghyoun Lee, Jing Xie, Hyeonjin Park, Hyun Jung, Jae-Min Oh

**Affiliations:** ^1^ Department of Chemistry, Dongguk University, Seoul 04620, Republic of Korea; ^2^ Department of Energy and Materials Engineering, Dongguk University, Seoul 04620, Republic of Korea

**Keywords:** deoxycholate, aggregation, layered double hydroxide, molecular arrangement

## Abstract

Deoxycholate (DA) is a natural emulsifying agent involved in the absorption of dietary lipids. Due to the facial distribution of hydrophobic-hydrophilic region, DA easily aggregates under ambient conditions, and this property hinders the practical application of DA in clinical applications. In this study, we found that the molecular arrangement of DA molecules could be controlled by using layered double hydroxide (LDH) under a specific reaction condition. The effect of reaction methods such as co-precipitation, ion exchange and reconstruction on the molecular arrangement of DA was investigated by X-ray diffraction, Fourier-transform infrared spectroscopy, high-resolution transmission electron microscopy and differential scanning calorimetry. It was demonstrated that the self-aggregation of DA molecules could be suppressed by the oriented arrangement of DA between the gallery space of LDH. The DA moiety was well stabilized in the LDH layers due to the electrostatic interaction between DA molecules and LDH layers. The most ordered arrangement of DA molecules was observed when DA was incorporated into LDH via a reconstruction method. The DA molecules arranged in LDH via reconstruction did not show significant exothermic or endothermic behaviour up to 400°C, showing that the DA moiety lost its intermolecular attraction in between LDH layers.

## 1. Introduction

Controlling molecular arrangement has attracted increasing interest in biology-related sciences as the molecular orientation affects various physicochemical properties in addition to the molecule's intrinsic properties. For instance, polymorphism, which refers to two or more crystalline phases of molecules, is one of the emerging issues in drug research. Due to the different intermolecular interactions and molecular arrangement, polymorphism influences various properties of drugs, such as dissolution, solubility, stability and hygroscopicity, drug efficacy, bioavailability, etc. [1–6]. Acetaminophen, one of the well-known painkiller drugs, was reported to show different compressibility behaviour during fabrication depending on the polymorphism [7]. Some drug molecules acquire additional thermodynamic stability due to the optimized intermolecular interaction [8,9]. It was also reported that the suppressed molecular interaction in a confined space resulted in a formation of a certain structure or a specific chemical reaction. Incorporation of amino acid molecules between the two-dimensional layered materials facilitated its polymerization [10,11]. Similarly, oligomerization of ribonucleotide was catalysed by the molecular arrangement in the interlayer space of clay material [12]. Furthermore, confinement of guanosine molecules in the interlayer space of layered metal hydroxide drove the formation of a certain supramolecular assembly through facilitated hydrogen bonding [13].

**Figure 1.**
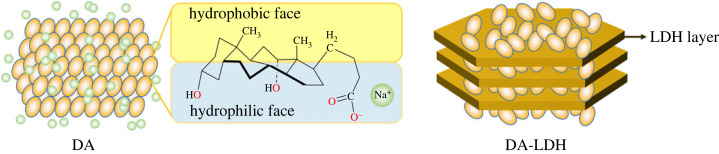
Schematic illustrations of DA crystal, molecular structure of DA and DA molecules arranged on LDH (DA-LDH).

One of the target substances to control molecular interaction for practical application is deoxycholic acid (DA). It is a naturally occurring amphiphilic molecule; in the living system, DA is found in bile acid and involved in the dissolution metabolism of fat. Due to its surfactant-like chemical nature and low toxicity, DA has been widely studied in cholesterol solubilization, dietary fat manufacturing, fat-soluble vitamin absorption, etc. [14–16]. Although DA has a versatile fat-dissolving role inside the biological system, its application *ex vivo* has been restricted due to its uncontrollable molecular interaction. Different from conventional chemical surfactants, which have a small polar head and a long hydrophobic tail, DA consists of a flat structure with hydrophilic and hydrophobic sides at each face (figure 1) [17,18]. Due to this structural property, DA sensitively forms aggregates at low pH, at improper temperature, or under imbalanced solvent conditions. In order to expand the application spectrum of DA, there have been several approaches to comprehend its molecular interaction. Like other surfactants, it has been reported that the solvent composition affected the molecular aggregation of DA. According to Das *et al*. the critical micellar concentration of DA increased—the molecular aggregation of DA tended to be prevented—as ethylene glycol was added to the aqueous DA solution [19]. As reported in other surfactants such as cetyltrimethylammonium bromide or tetradecyltrimethylammonium bromide, [20] the addition of low surface tension solvent can reduce the molecular interaction, resulting in increased critical micellar concentration. On the other hand, the existence of a certain additive was known to accelerate aggregation of DA moiety. Amino acids, such as lysine, arginine and histidine, were reported to reduce the critical aggregation concentration of DA [21]. It was inferred that both electrostatic and hydrophobic interactions between DA and amino acids collect the DA moieties more tightly to facilitate molecular aggregation. Incorporation of DA with layered inorganic materials such as layered double hydroxide (LDH) can be suggested as a strategy to control the aggregation of DA, allowing effective, versatile application of DA. There have been several approaches to incorporating DA moiety into LDHs; however, most of the studies used complicated or energy-consuming synthetic routes. For example, Ogawa *et al*. intercalated deoxycholic acid into LDH through a hydrothermal reaction, resulting in the topotactically expanded particle thickness of LDH toward c-axis [22]. On the other hand, Wu *et al*. intercalated DA into LDH nanohybrids via exfoliation of LDH and the co-assembly arrangement of LDH and DA [23]. Both studies were successful in preparing DA intercalated LDH; however, the DA moiety is inevitably subjected to harsh physical and chemical circumstances during synthesis. Hydrothermal condition accompanies high temperature and pressure; the organic moiety encounters formamide—a strong amphiphilic solvent—during the exfoliation-reassembly process. Our purpose is to find the most effective and simple way to accommodate DA moiety into LDH through general synthetic routes such as coprecipitation, ion exchange or reconstruction.

In this study, we are going to suggest a method to control the molecular arrangement of DA using inorganic material. It was previously reported that the organic molecules could be aligned at the surface of inorganic materials, possibly through adsorption or electrostatic interaction, and that the preferred orientation of molecules can alter the physicochemical properties of the molecules. For instance, methyl orange dyes were incorporated in the interlayer space of layered metal hydroxide to have modified excitation-emission wavelength [24]. Electrostatic interaction between molecules and inorganic particles arranges the organic molecules to control the light absorption-emission property of luminescent species [25]. Inorganic particles could also stabilize vulnerable organic molecules to make bulk-like stability but single-molecule-like photophysical properties [26]. In addition, the confinement of benzene molecules in the pore channel of MCM-41 reduced its diffusion coefficient due to the modified molecular orientation [27]. Taking into account the previous research, we tried to incorporate DA molecules in the interlayer space of LDH. The LDH, of which chemical formula is expressed as [M(II)_1-x_M(III)_x_(OH)_2_]^x+^ (A^n−^)_x/n_, (M: metal, A: anion) consists of positively charged nanolayers and electrostatically stabilized interlayer anions [28–30]. As the LDH layer provides periodic positive charge as well as large layer, we hypothesized that the incorporation of DA into LDH resulted in the arrangement of molecules in a two-dimensional and controlled manner, which is illustrated in figure 1. In addition, the molecular interaction, which was governed by the inter-molecular van der Waals force, would be regulated by the DA-LDH electrostatic interaction. The point of this study is how to incorporate DA into LDH by overcoming the strong inter-molecular interaction. In order to answer this question, we approached the three well-known intercalation methods in LDH, coprecipitation, ion-exchange and reconstruction. The interaction between DA molecules and LDH layers, depending on the incorporation methods, will be discussed in terms of crystal structure, chemical bonding, microstructure and molecular interaction.

## 2. Material and methods

### 2.1. Material

The DA sodium salt with more than 98% purity was purchased from Sigma-Aldrich LLC, MO, USA. Magnesium nitrate hexahydrate (Mg(NO_3_)_2_·6H_2_O), aluminium nitrate nonahydrate (Al(NO_3_)_3_·9H_2_O), sodium hydroxide (NaOH), sodium bicarbonate (NaHCO_3_) and sodium nitrate (NaNO_3_) were all purchased from Daejung Co. Ltd., Korea. All the reagents were used as purchased.

### 2.2. Incorporation of DA into LDH

In order to incorporate DA into LDH through coprecipitation (DL-C), 0.015 mol of DA was first dissolved in 200 ml of decarbonated water. Then, a solution containing 0.03 mol of Mg(NO_3_)_2_·6H_2_O and 0.01 mol of Al(NO_3_)_3_·9H_2_O was added to the DA solution. The mixture was then titrated with 1 mol l^−1^ of NaOH solution to reach a pH of approximately 9.5. The reaction vessel was kept for 1 day under nitrogen bubbling in order to avoid undesired contamination by atmospheric carbon dioxide. The white precipitate was separated by centrifugation and washed with decarbonated water three times.

For the synthesis of DA-LDH through ion exchange (DL-I), the pristine LDH was prepared in advance. An aqueous solution containing 0.2 mol l^−1^ of total metal concentration (ratio of Mg(NO_3_)_2_·6H_2_O over Al(NO_3_)_3_·9H_2_O was set 3) was prepared in decarbonated water and titrated with 1 mol l^−1^ NaOH solution up to pH approximately 9.5. In order to avoid carbonate contamination, the reaction was carried out under nitrogen bubbling, and NaNO_3_ solution (0.12 mol l^−1^, 100 ml) was added during titration. The white suspension was kept for 1 day under vigorous stirring and nitrogen bubbling. The white precipitate, MgAl-NO3-LDH, was collected by centrifugation and washed with decarbonated water three times. The precipitate was then resuspended in decarbonated water and mixed with DA solution (0.1 mol l^−1^). The molar ratio of Al in LDH and DA was set at 1 : 3 (Al : DA). The mixed suspension was incubated for 1 day under vigorous stirring and N_2_ bubbling. The final precipitate was separated by centrifugation and washed with decarbonated water three times.

The DA-LDH made by reconstruction (DL-R) was prepared as follows. First, MgAl-CO3-LDH was prepared as described in MgAl-NO3-LDH. Both decarbonated water and sodium nitrate were replaced by deionized water and sodium bicarbonate, respectively, for the MgAl-CO3-LDH synthesis. The lyophilized MgAl-CO3-LDH was thermally treated in a muffle furnace at 600°C for 12 h. Thus, obtained metal oxide (0.5 g) was dispersed in a DA solution (0.0.05 mol l^−1^, 200 ml). The mixture was reacted for 1 day under N_2_ bubbling. The final precipitate was separated by centrifugation and washed with decarbonated water three times.

All three DL hybrids were lyophilized and ground in an agate mortar to get fine powder.

### 2.3. Characterization

Structural characterizations for DA and LDHs with DA were carried out with X-ray diffraction (XRD) and Fourier-transform infrared (FT−IR) spectroscopy. The XRD patterns were collected in the 2*θ* range from 5° to 80° with time-step increments of 0.02° and 0.5 s per step. The XRD patterns were obtained with Ultima IV (Rigaku, Tokyo, Japan) by using Ni-filtered Cu–K*α* radiation (*λ* = 1.5409 Å, 40 kV, 30 mA) as an X-ray source. The FT−IR spectra were recorded using an FT/IR-4600 spectrometer (JASCO, Tokyo, Japan) in the range (2000–600) cm^−1^, with 4 cm^−1^ resolution, to detect transmission at room temperature. The spectrometer was equipped with an attenuated total reflectance (ATR) accessory and Ge crystal. The powders of DA and LDH with DA were placed on the crystal and directly measured via ATR. The morphology of the DA and LDH with DA were visualized by scanning electron microscopy (SEM) with JSM-6700F (JEOL, Tokyo, Japan). The measured sample was prepared by carefully spreading the powder on carbon tapes. Particle sizes, morphologies and the crystallographic arrangement of the LDHs were observed under field emission transmission electron microscope (FE-TEM: JEM-F200, JEOL) with an accelerating voltage of 200 kV. The TEM samples were prepared by placing drops of ethanol suspension of LDHs onto carbon-coated copper grids with a 200 mesh. After the sample settlement, the grids were dried in air to remove the solvent for 1 h. Thermal analysis was conducted using differential scanning calorimetry (DSC) of Jade DSC (PerkinElmer, Inc., WA, USA) set for scanning from 20 to 400°C at 10°C/min under nitrogen atmosphere. Hydrodynamic radii of LDH and DA-LDH suspension at a concentration of 1 mg ml^−1^ in water were measured by dynamic light scattering (DLS) with ELSZ-1000 (Otsuka electronics, Hirakata, Japan).

## 3. Results and discussion

### 3.1. Crystal structure

**Figure 2.**
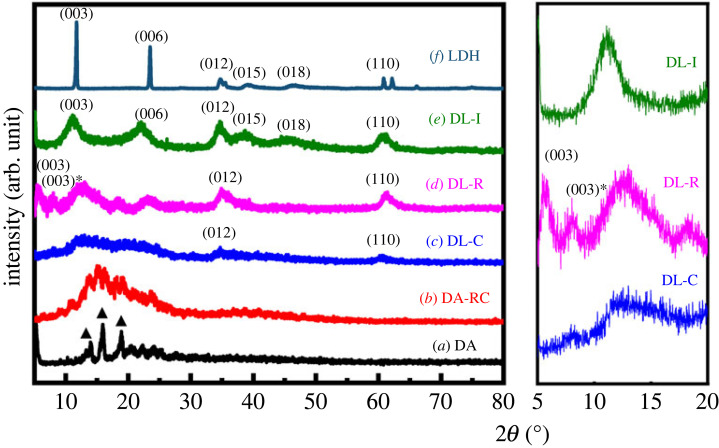
Powder X-ray diffraction patterns of (*a*) DA, (*b*) DA-RC, (*c*) DL-C, (*d*) DL-R, (*e*) DL-I and (*f*) LDH. The right panel is a magnified diffractogram of three DA-LDH hybrids, DL-C, DL-R and DL-I, in low angle region. The two peaks of DL-R in low angle regions may be two (003) peaks from different interlayer spaces; they are denoted as (003) and (003)* for clarification.

The crystal structure of three DA-LDH and DA only were investigated with powder X-ray diffraction, as shown in figure 2. It was clearly demonstrated that the sodium salt of DA had a crystalline structure (figure 2*a*) as reported in the previous literature [31,32]. It was known that the crystalline DA became amorphous by dehydration and recovered crystallinity upon appropriate hydration [31]. The fact suggests that the aggregation of DA molecules is fairly strong and reversible in the existence of water molecules. We also observed aggregation and crystallization of DA after sequential dissolution and precipitation. Sample DA-RC, of which XRD pattern was represented in figure 2*b*, was prepared by treating Na-DA solution with 1 mol l^−1^ hydrochloric acid. The clear Na-DA solution readily turned to white precipitates along with crystallization of molecules. Although the X-ray diffractogram of DA-RC was generally amorphous (figure 2*b*), it still exhibited representative peaks of DA crystal in the 2*θ* range 10–20°.

The sharp crystalline peaks, which are typical features of salt, became broad upon reaction with LDH. The hybrid obtained by direct coprecipitation (DL-C) showed almost amorphous diffraction pattern with two small peaks at around 2*θ* = 35° and 60° attributed to (012) and (110) reflection of LDH lattice (figure 2*c*). The absence of (00l) peaks in the diffractogram of DL-C, as well as the small lattice peaks, suggested that small LDH crystallites were precipitated in the presence of DA. Although we could not observe the evidence that DA molecules were intercalated into LDH layers in DL-C, it could be at least proposed that small LDH crystallites restrain inter-molecular crystallization of DA moiety. The figure 2*d* and the corresponding magnified diffractogram for DL-R—the hybrid obtained by reconstruction—clearly showed both (00l) peaks and lattice peaks of LDH. DL-R was obtained by the calcination of the LDH intercalated with carbonate anions and reconstruction of LDH in the existence of deoxycholate anions. It was noteworthy that DL-R exhibited two peaks in the low angle region, at 2*θ* values of 5.5° and 8.1°, respectively. Compared with pristine LDH (figure 2*f*), the (003) peaks shifted from 11.3° to the lower angle region, indicating that the deoxycholate anions were located in the interlayer gallery of LDH. In addition, the d-spacing of the two peaks at 2*θ* values of 5.5° and 8.1° corresponded to 1.60 nm and 1.09 nm, respectively. It was noteworthy that DL-R exhibited two peaks in low angle region, at 2*θ* value 5.5° and 8.1°, respectively. The d-spacing of the two peaks corresponded to 1.60 nm and 1.09 nm, respectively. The two peaks can be interpreted as a set of (00l) peaks—(006) and (009)—or two (003) peaks from different interlayer spacings. The former interpretation hypothesized (003) peak at 2.7° with the d-spacing of 3.21 nm, possibly forming double layer orientation of DA in the interlayer space. According to the latter interpretation, two different d-spacings of 1.60 nm and 1.09 nm existed in the sample depending on the tilting angle of DA moiety in a single-layer manner. The discussion on the interlayer arrangement will be clarified in the high-resolution transmission electron microscopy part. In either case, the interlayer space expanded sufficiently large to accommodate DA molecules between LDH layers. Unfortunately, the DL-I, the hybrid prepared by ion exchange reaction, did not show any evidence of DA intercalation. The diffractogram only exhibited signals from pristine LDH, suggesting that DA moieties were simply adsorbed at the surface of LDH rather than incorporated into LDH on a nanometer scale. It could be therefore concluded that either coprecipitation or reconstruction was effective in getting homogeneous combination of DA and LDH in the nanoscopic dimension. Between the two methodologies, reconstruction was determined to be the more efficient for separating DA molecules by using nanospace of LDH interlayer; two-dimensional arrangement of DA molecules in DL-R would restrict their molecular aggregation.

**Figure 3.**
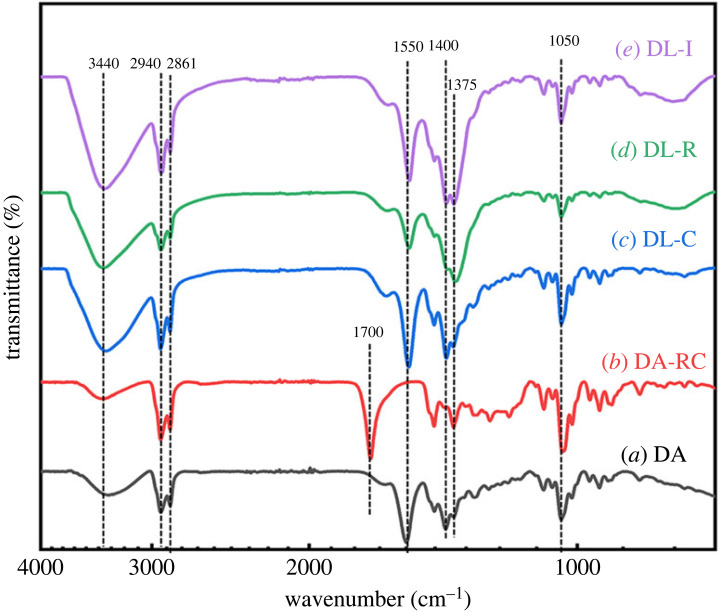
FT-IR spectra of (*a*) DA, (*b*) DA-RC, (*c*) DL-C, (*d*) DL-R and (*e*) DL-I.

In order to further inspect the DA moiety incorporated with LDH, the chemical structure of DA moiety with LDH at each state was examined by FT-IR spectroscopy. The chemical structure of DA moiety at each state was examined by FT-IR spectroscopy. As shown in figure 3, all the materials showed characteristic peaks of DA backbone at around 1375 cm^−1^ and 1050 cm^−1^, which attributed to –CH_3_ and –CCO^−^ functional groups, [23,33,34] suggesting that the intact structure of DA did not change upon recrystallization nor hybridization with LDH. The wide band at 3440 cm^−1^ for all the samples was attributed to the symmetric and antisymmetric O–H stretching modes of DA and interlayer water molecules of LDH or surface absorption water [35–37]. It could also be noted that the asymmetric and symmetric methylene stretching bands of DA were 2940 cm^−1^ and 2861 cm^−1^, respectively [35,38]. Different from the other samples, DA-RC, which is the recrystallized DA upon acid treatment, showed clear peaks at around 1700 cm^−1^ due to the existence of carboxylic acid (–COOH) groups. It was expected that the sudden addition of hydronium ion in Na-DA solution gave rise to protonation of carboxylate moiety, reducing the quantity of Na^+^–COO^−^ ionic bond. As expected, all the four spectra except DA-RC showed two peaks at around 1550 cm^−1^ and 1400 cm^−1^, which was originated from antisymmetric and symmetric stretching of carboxylate (–COO^−^) moiety, respectively [39–41]. The results suggested that all the three hybrids contained DA molecules and that the DA well preserved their carboxylate moiety, facilitating the charge-charge interaction between DA (negative charge) and LDH layers (positive charge), as suggested in the schematic diagram in figure 1.

### 3.2. Particle morphology

**Figure 4.**
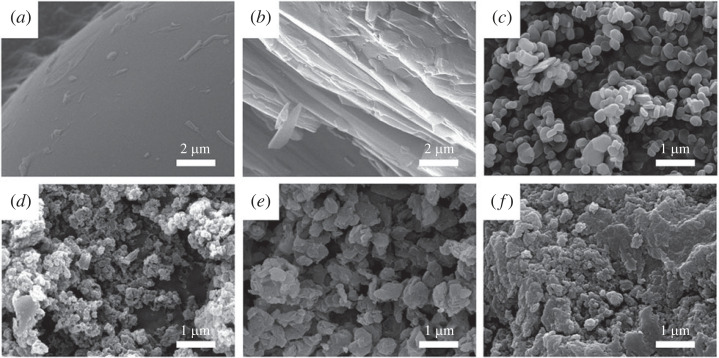
SEM images of (*a*) DA, (*b*) DA-RC, (*c*) LDH, (*d*) DL-C, (*e*) DL-R and (*f*) DL-I.

The overall morphology of the samples was examined on a microscopic scale. As shown in figure 4*a*, the DA itself showed very large lumps of particles, of which the surface was smooth. The large lump is strongly related to the high crystalline phase of DA in XRD (figure 2), indicating that the intermolecular aggregation was strong. The recrystallized DA (DA-RC) also showed large lumps; however, the feature was fairly different from DA alone (figure 4*b*). It was recently reported that the Na-DA could aggregate in various forms, including rods, sponges, vesicles, lamellae, bundles of tubes, etc. [42]. Similar to the conventional surfactants, which form micelle, hexagonal array and lamellar structure depending on the concentration, the DA molecules gathered into lamellar or tubular morphology. In the process of DA-RC formation, the rapid increase in hydrophobicity might accelerate the strong intermolecular interaction through one-dimensional or two-dimensional direction, to form such a lamellar or needle-like morphology. The SEM images of three hybrids were different from that of DA or DA-RC. All the samples showed particles of less than 1 µm, which corresponded to the dimension of LDH particles (figure 4*c*). This clearly showed that the crystalline nature of DA was highly suppressed after the reaction with LDH, although the detailed feature of DA would be different according to the hybridization method. As shown in figure 4*d*, the DL-C, the coprecipitated hybrid, showed smaller particles compared with the other hybrids. The small particle size was attributed to the low crystallinity, as shown in the XRD (figure 2*c*). The DL-R had a relatively larger particle size than the other hybrids and did not show specific morphology (figure 4*e*). This is a typical morphology of LDH after reconstruction with organic moiety [43–45]. As the layer-by-layer stacking is slightly disordered in the reconstruction reaction, the particles of organically reconstructed LDH tended to show a random assembly of lamellar sheets. The SEM image of DL-I exhibited relatively inhomogeneous and irregular particles (figure 4*f*). Considering that the DL-I showed crystalline peaks corresponding to LDH (figure 2*e*), the small size and irregularity of particle in SEM image are not expected features. The discrepancy can be explained by the surface-adsorbed organic moieties. The LDH particles did not accommodate DA moiety in their interlayer space but adsorb at the surface. The DA molecules then aggregated on the LDH surface to camouflage the morphology of LDH. The organic moiety at the surface of LDH hides the crystalline particles, resulting in the irregular surface morphology.

**Figure 5.**
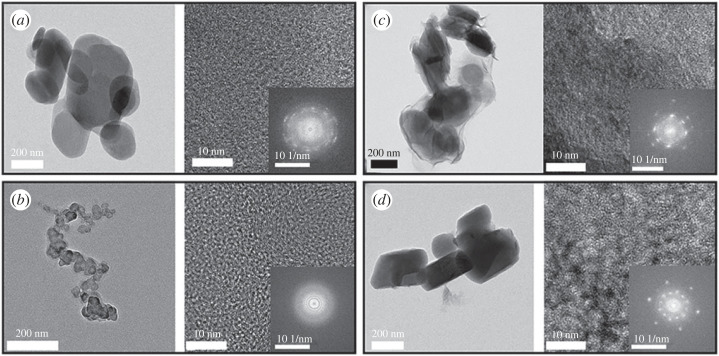
TEM images and corresponding fast Fourier transforms (FFT) patterns of (*a*) LDH, (*b*) DL-C, (*c*) DL-R and (*d*) DL-I.

**Figure 6.**
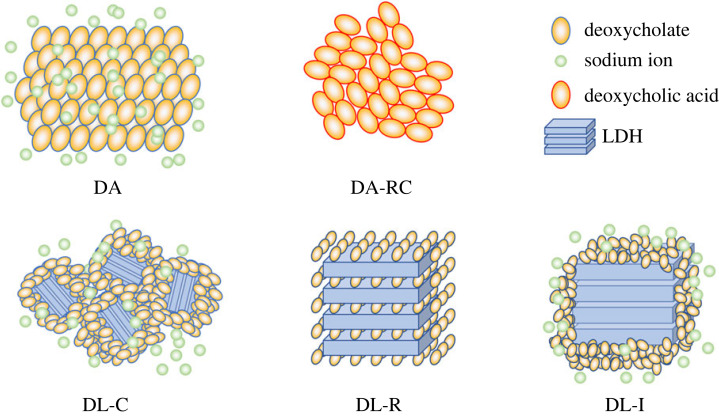
Schematic structures of DA powder (DA), recrystallized DA (DA-RC), and the three hybrids obtained by coprecipitation (DL-C), reconstruction (DL-R) and ion-exchange (DL-I).

In order to investigate the intracrystalline structure of DL hybrids in detail, transmission electron microscopy (TEM) was carried out to obtain images and fast Fourier transform (FFT) patterns (figure 5). As shown in the electronic supplementary material, figure S1, the organic moiety, such as DA itself and recrystallized DA (DA-RC), were unstable under electron beam irradiation; the TEM did not show images of typical crystalline material. The particle sizes of LDH, DL-C, DL-R and DL-I were statistically measured by selecting at least 20 particles from TEM images and compared through Student *t*-test (electronic supplementary material, figure S2 and table S1). The mean particle sizes of LDH, DL-C, DL-R and DL-I were calculated to 219 ± 63 nm, 34 ± 8 nm, 213 ± 46 nm and 218 ± 70 nm, respectively, revealing that there was no significant particle size difference among LDH, DL-R and DL-R. The result suggested that the reactions in ion exchange and reconstruction occurred in a topotactical manner maintaining particle diameter. The reason why DL-C showed significantly small particle size was the different synthetic approach. In the coprecipitation, the LDH particles were grown *in situ* with the arrangement of DA molecules. The small particle size of DL-C corresponded to its low crystallinity reflected by the ring pattern in FFT. The pattern represented that the DL-C hybrids had amorphous-like structure for both inorganic (LDH) and organic (DA) moiety. This result corresponded to the XRD pattern (figure 2*c*), in which only a few crystalline peaks corresponding to the lattice of LDH were found. The DL-R showed a well-defined image of particles. Although the particle size and the particle shape of LDH and DL-R were similar, there were no distinct particle edges of DL-R shown in figure 5*c* and electronic supplementary material, figure S3, proposing that the highly ordered-layered structure of LDH was absent after reconstruction attributed to the intercalation of DA moieties. As we discussed in the SEM part, the fluffy moiety is the LDH layer that lost the ordering of layer-by-layer stacking during reconstruction process. Based on the morphological change and the clear hexagonal spot patterns in FFT of DL-R (figure 5*c*), we could expect that the LDH layers were maintained and the organic/inorganic hybridization homogeneously occurred at a nanoscopic level. The TEM image of DL-I showed a typical feature of inorganic particles except for the existence of a grey contrast part outside of the particle. Furthermore, the FFT pattern of DL-I was almost same as that of conventional LDH, suggesting that there was no change in LDH part after the reaction. Thus, we expected that the ion exchange reaction was not enough to induce organic-inorganic nanohybrid and that the inorganic part (LDH) and organic part (DA) existed with phase separation. To summarize, the DL-R hybrid had the most periodic hybridization between DA and LDH in nanoscale among the three hybrids. In addition, the DL-C had an amorphous mixture of DA and LDH, which may not take advantage of LDH layers to the direct molecular arrangement. Further, the DL-I did not induce significant molecular arrangement change of DA moiety. Taking into account the XRD, SEM and TEM results, we could suggest the schematic diagram of DA arrangement as illustrated in figure 6. The crystal of DA had highly ordered arrangement DA, while the recrystallized DA (DA-RC) had aggregation with relatively low crystallinity due to the sudden growth of crystal. In the three DL hybrids, both DA-C and DA-I did not successfully accommodate DA moiety into the structure but adsorbed DA on the surface. On the other hand, DL-R is considered to arrange DA moiety in the most ordered manner by confining the organic moiety in the two-dimensional space.

### 3.3. Interlayer arrangement

**Figure 7.**
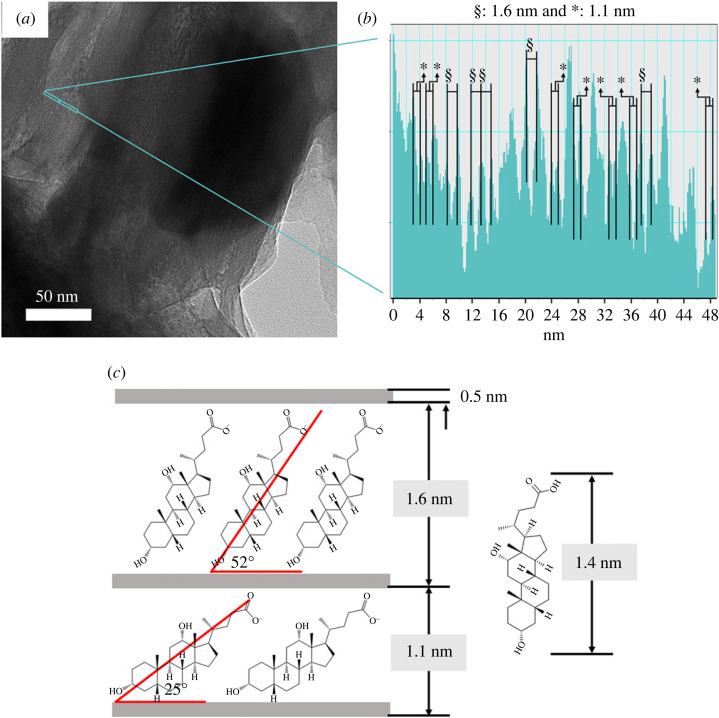
(*a*) TEM image and (*b*) the histogram obtained from a green rectangle in TEM for visualizing interlayer distance of DL-R. (*c*) Schematic illustration on the interlayer arrangement of DA moiety in LDH.

As it was suggested by the XRD and TEM that DL-R hybrid has the most ordered orientation of DA moiety along the ab-plane among the DL hybrids, we further investigated the layer-by-layer arrangement of the hybrid by using HR-TEM and electron density histogram. Figure 7*a* exhibited the magnified image of a DL-R hybrid, which happened to orient perpendicular to the *c*-axis. We could clearly observe the assembly of lines, which was attributed to the layer stacking of LDHs. The electron density histogram along the *c*-axis, which was taken from a set of line assemblies in HR-TEM image, was displayed in figure 7*b*. The electron density peaks were regularly arranged with 1.6 nm or 1.1 nm distances. From the XRD patterns, we expected either (i) a double-layer arrangement of DA with 3.21 nm d-spacing or (ii) a single-layer arrangement with two different tilting states with 1.60 and 1.09 nm. The electron histogram clearly showed that the latter interpretation was correct. As suggested in figure 7*c*, DA molecules can be arranged in the interlayer space with tilted or horizontal orientation, depending on the intermolecular attraction and the organic-inorganic interaction. Since LDHs have positively charged metal hydroxide layers and anions in the interlayer gallery, DA molecules as anionic organic species could interact with LDH by periodic electrostatic affinity when a proper synthetic condition is applied [22,23].

**Figure 8.**
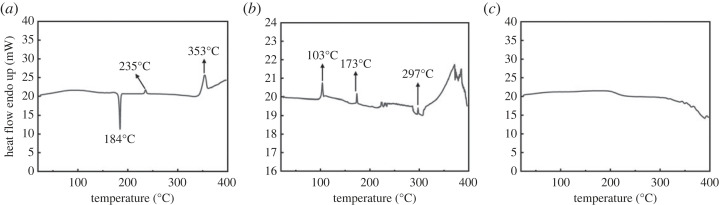
DSC curves of (*a*) DA, (*b*) DA-RC and (*c*) DL-R.

As we confirmed that the three-dimensional assembly of DA was rearranged to the two-dimensional direction in the DL-R hybrid, we further investigated the intermolecular attraction in DA moiety using the DSC technique. The DA as it is showed one distinct exothermic peak at 184°C and two endothermic peaks at 235 and 353°C, respectively (figure 8*a*). According to the previous literature, deoxycholic acid showed various behaviours with respect to temperature [46–49]. Some papers reported the endothermic behaviour of DA at around 200°C due to melting; [46,47] other papers observed step-by-step exothermic and endothermic behaviours upon temperature increase [48]. Different thermal behaviours of DA may be attributed to the various molecular arrangements resulting from the strong intermolecular interaction. As reported previously, [49] the exothermic peak at around 180°C was attributed to the recrystallization, and the endothermic reactions were related to the intermolecular separation­—melting. The DSC diagram of recrystallized DA-RC was slightly different from that of DA alone. As displayed in figure 8*b*, we could observe sharp endothermic peaks at 103°C, 173°C, 297°C and a broad endothermic phenomenon at around 350°C. The first endothermic reaction at 103°C was attributed to dehydration, as the sample contained lots of water moiety during the recrystallized process. The sharp endothermic peaks of DA-RC at 173°C and 297°C corresponded to those of DA alone at 184°C and 235°C. As the recrystallization was carried out rapidly, the DA molecules did not have enough time to fully aggregate themselves. Thus, the molecular separation—melting—of DA-RC occurred at a lower temperature than DA alone. Anyway, both DA and DA-RC, which are thought to have molecular interaction in a three-dimensional direction, exhibited discrete exothermic or endothermic behaviours due to the active intermolecular attractions. On the other hand, the DSC diagram of DL-R, the reconstructed DA-LDH hybrid, did not show heat flow until 350°C (figure 8*c*). This result implied that there was restricted molecular interaction of DA in the DL-R hybrid. The molecular arrangement of DA between the LDH layers was more governed by the charge-charge interaction between DA and LDH than the molecule-molecule interaction. It is therefore concluded that the strong and uncontrolled molecular aggregation of DA could be suppressed through molecular rearrangement in between the LDH layer by the reconstruction reaction.

**Figure 9.**
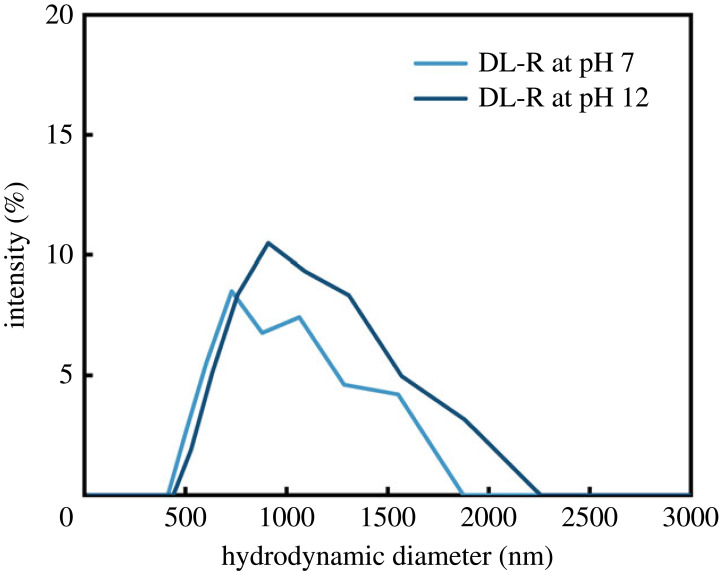
DLS size distribution by intensity of the DL-R prepared at pH 7 and pH 12 in water and pH adjusted by using 1 M of NaOH.

The stability of DL-R in both neutral (pH 7) and basic (pH 12) media was monitored through DLS. The hydrodynamic diameters of DL-R in pH 7 aqueous condition presented a similar particle size distribution to that of DL-R in pH 12 aqueous suspension, as shown in figure 9. The particle sizes of DL-R remained unchanged even under strong base conditions, as the LDH protected the payloads from basic condition. As the LDH moiety can be soluble in acidic media, the colloidal stability of DA-LDH in acidic condition may be different from that in neutral pH. However, it can be suggested that the dissolution of LDH in acidic media plays a buffering role in protecting DA moiety from the attack of acid [50,51].

DA has been used to reduce submental fat [52,53] and play a role in the emulsion as emulsifiers [54,55] to enhance the stability of immiscible liquids. However, there have been detrimental issues of DA, such as strong aggregation in certain media, resulting in the loss of functionality. The incorporation of DA in the LDH interlayer space could avoid molecular aggregation due to the formation of an inorganic layered network surrounding DA molecules. Once stabilized DA can effectively perform its inherent functions, such as an emulsifier, a fat dissolver, etc. in a variety of media. Indeed, the DA-LDH could be used in the formulation of oil in water emulsion [50], which can be further used as cosmetics for transdermal delivery platforms.

## 4. Conclusion

The DA molecules were incorporated into the interlayer space of LDH through three synthetic strategies in order to control the molecular arrangement. Conventional coprecipitation resulted in the development of small crystallites of LDH, resulting in the coexistence of both LDH particles and DA crystals. Although the crystallinity of DA decreased after coprecipitation with LDH, the method was not efficient in arranging DA molecules in a preferred manner. The ion-exchange reaction between the DA solution and MgAl-NO3-LDH resulted in no change in crystal structure, resulting in the surface adsorption of DA moiety. The crystallinity of DA decreased after the ion-exchange reaction; however, we did not consider this method practical as the surface adsorbed amount would be very low. On the other hand, the reconstruction method, in which DA solution and metal oxide reacted together, gave rise to the well-ordered DA moieties in between the LDH layers. The X-ray diffraction and FT-IR spectroscopy indicated that the DA molecules were well stabilized through electrostatic interaction between DA and LDH and that the molecules were tilted by 25° or 52°, minimizing intermolecular interaction over DA-LDH attraction. The HR-TEM images and electron-density line profile clearly showed that the DA molecules were preferentially arranged in two-dimensional direction when DA and LDH were hybridized through the reconstruction method. Finally, the DSC curves confirmed that there was no significant intermolecular attraction among DA, when the molecules were incorporated into LDH through the reconstruction reaction.

## Data Availability

Data that support this study have been uploaded as part of the electronic supplementary material [56]. In addition, the datasets can also be found at https://doi.org/10.5061/dryad.q2bvq83qk [57].
